# Supplementary material for: Developing a real-time hand-gesture recognition technique for wheelchair control
Source: PLoS One. 2025 Apr 24;20(4):e0319996. doi: 10.1371/journal.pone.0319996 (PMC12021275; doi:10.1371/journal.pone.0319996)
Supplement: Attachment — Submitted filename: Response to Reviewers Comments.docx [file pone.0319996.s002.docx]

**RESPONSES TO REVIEWERS’ COMMENTS**

We would like to thank the reviewers for their careful read and constructive feedback on the previous draft entitled “**Developing a Real-Time Hand-Gesture Recognition Technique for Wheelchair Control**”. We have carefully considered their comments in preparing our revision, which has resulted in a manuscript that is clearer, more compelling and broader.

**Reviewers' Comments:**

....................................................................................................................................................................

**Reviewer #1:**

**Comment #1:** *Please add your contributions in bullet points for clarity in the Introduction section.*

**Response:** Thank you for your humble consideration and recommendation. The contributions of our paper have been added in bullet points in the Introduction section. [Please see Introduction (Section 1) in page 4 of the revised manuscript].

Contributions of this research are -

- A unique mathematical model has been designed utilizing hand landmarks and some thresholds for hand gesture recognition
- An efficient, flexible gesture recognition system for the control of a wheelchair has been proposed which requires only movement of fingers
- A better gesture recognition system for wheelchair control has been proposed focusing these aspects all together: users’ flexibility, high accuracy, and environment (indoor and outdoor) independency.

**Comment #2:** *It is better to use the same convention to present the same thing. For example, “Tejonidhi et al. [24]” and “Sadi, M.S. et al. [25]” should follow the same presentation style.*

**Response:** Thank you for your thoughtful observation. The mentioned presentation style has been corrected in revised manuscript and same convention has been used to present the same things. [Please see Related Work (Section 2) in page 6 of the revised manuscript].

Sadi et al. [25] designed a wheelchair system which is controlled by hand gestures in real time, where the authors used YCrCb skin segmentation technique, Haar-cascade classifier, KCF tracking algorithm, and a 2D CNN model.

**Comment #3:** *Please provide a summary table for the works mentioned in the Related Work section. This addition would give an in-brief overview of the existing state-of-the-art.*

**Response**: Thank you for your insightful comment and recommendation. A summary table with overview of related works has been added in revised manuscript. [Please see Table 1 under Related Work (Section 2) in page 7 of the revised manuscript].

**Table 1. An Overview of Related Work.**

| **Existing Technique** | **Functionality** | **Limitations** | **Major Equipment** | **Accuracy** |
| --- | --- | --- | --- | --- |
| Gao et al. [19] | Hand gesture recognition | Requires hand raising, background complexity | Microsoft Kinect Camera, highly configured laptop | 10-100% (Depends on background complexity) |
| Oliver et al. [21] | Hand movement detection | Requires wearing hand band | Accelerometer, Joystick Manipulator | Actual accuracy is not measured |
| Sadi, M.S. et al. [25] | Hand gesture recognition | Requires finger movement, doesn’t work well under daylight or if the background and skin tone match | RGB Camera, Raspberry Pi | 97.14% |
| Megalingam et al. [18] | Finger movement detection | The user must navigate the touchscreen with their hands or fingers and know how to operate the application. | Arduino Uno, HC05, Bluetooth, Android device | 98% |
| Desai et al. [20] | Eye movement detection | It’s a preliminary work to model the  control of a wheelchair, creates complication in real-time | HD Camera, MATLAB. | Actual accuracy is not measured |
| Kutbi et al. [22] | Head movement Tracking | User’s head-motion is detected through a head-mounted egocentric camera, which is not user-friendly. | An egocentric camera, TI-TAN18CS, Arduino Mega, ROS | 85.7% |
| Mahmud et al. [23] | Multi-modal wheelchair control mechanism | It’s integration of a joy stick with hand  gesture, eye gaze and head movement tracker. Not user-friendly, tracking sensors must be attached to the user's body | Raspberry pi, RGB camera, a modified VGG-8 model, and an accelerometer | 90% |
| Tejonidhi et al. [24] | Eye pupil’s movement detection | Eye pupil is tracked by a camera and the system glows LEDs based on eye movements. The detection procedure is crucial for real-time applications | Web camera, Philips microcontroller and the Viola-Jones MATLAB algorithm | 70% to 90% |

**Comment #4:** *I noticed that both “Experimental Setup” and “Results” started with the numbering of 4.1. Are they correct?*

**Response**: Thank you for your precise observation. Sorry for the inconvenience. The Section numbering of “Results” section has been corrected in revised manuscript. [Please see “Results” in page 18 of the revised manuscript].

**Comment #5:** *The work presented in [27] obtained an accuracy of 99.17%, whereas this work achieved an accuracy of 98.14%. Also, many results are presented in this work from [27]. Please explain in detail the superiority of this work over [27].*

**Response**: Thank you for your incisive statement and observation. The methodology used in the work presented in [27] is different from the proposed new methodolgy in this paper. In the previous work of [27], all the thresholds were considered as fixed values but in the new proposed methodology, a range of maximum and minimum threshold values have been used which has made the system more dynamic, accessible and it showed better performance. Though the accuracy in the work presented in [27] was higher, but sample count was very low. In the work [27], during performance evaluation, only 20 samples were taken for each gesture, which was not reflecting the actual efficiency of the system as it considered lower variety of sample. Whereas, in the proposed new methodology, 100 samples were used for each gesture for evaluating performance which gives better reflection. For fair comparison, the old methodology was also tested for similar number of samples (100 per gesture) and found accuracy is lower (97%), whereas, the proposed new methodology attained higher accuracy (98.14%). So, comparing fairly, the proposed new methodology is performing better than the previous one. The confusion matrix for old methodology of [27] for similar number of samples has been provided in the manuscript. [Please see page 22-25 of the revised manuscript (under section 4.2)].

There is an improvement in this work compared to our previous work [27] on the same topic. In our previous work, all the thresholds were considered as fixed threshold values rather than a range of maximum and minimum threshold values. Moreover, those thresholds were not automatically calculated from hand samples. That is why the technique was less dynamic and sometimes the output was misleading. Again, the technique was not working good for some special disability cases. For example, for the ‘Drive’ gesture, detection was not possible for those users who cannot make his index finger fully straight for any illness or disabilities. Hence, the distance between wrist to fingertip didn’t meet the condition with threshold. Keeping this in mind, a maximum and minimum values were determined for all thresholds at the beginning of hand detection for once. After using this range concept, it would be easy to meet the thresholds and making the technique more dynamic and more useful for disabled people. Fig. 8 to Fig. 11 illustrates these issues and comparison between our old and new methodology.

**Comment #6:** *I did not find any differences between a few of the figures presented in [27] and this work. The figures are Fig. 1, Fig. 2, Fig. 4 and Fig. 5 [27], Fig. [5], and Fig. 6 [27]. Could you please clarify these things? .*

**Response**: Thank you for your intuitive observation. Fig. 1 of the manuscript contains the major steps of the hand gesture recognition technique which is same for [27] but the methodology, thresholds calculation and samples count is different in these two works. Fig. 2 represents the positions and names of the major hand landmarks in a hand which is universally used for the framework used for hand gesture recognition in the two works which is used for explaining the steps. Fig. 4, Fig. 5 [27], Fig. [5] and Fig. 6 [27] contains the classes of hand gestures used in the proposed technique for performing different operations of wheelchair (Stop, Horn, Drive). Same gestures has been used in the proposed new methodology for fair comparison and performance evaluation.

**Comment #7:** *Please use the same reference style for all references. For example, [15], [16], [21]-[23], and [25]-[26] have been deviated from other reference style. It is better to check all the references again to add the missing volume, page number, issue, etc.*

**Response**: Thank you for your important observation and comment. Sorry for the inconvenience. Reference styles have been corrected where required and same reference style have been used for all references in the revised manuscript.

**Comment #8:** *Please add a picture of the actual users, operating the wheelchair.*

**Response**: Thank you for your suggestion and recommendation. The picture of actual users, operating the wheelchair have been added in the revised manuscript. [Please see Fig. 9 at page 19 of the revised manuscript (under section 4.1)].

**Reviewer #2:**

**Comment #1:** *The method presented is dedicated to individuals with major motor disabilities who cannot properly use a joystick. In the vast majority, these individuals cannot properly use their fingers either. The study appears to be conducted on a sample of able-bodied individuals. A qualitative study conducted on individuals with disabilities is lacking.*

**Response**: Thank you for your insightful observation and comment. The methodology presented in the manuscript will be applicable only for those disabled persons who can move only their fingers of one hand. Operating the joystick requires more energy and muscle power than moving only fingers. The proposed methodology has also been tested by a sample individual with disabilities. [Please see Fig. 9 at page 19 of the revised manuscript]. A qualitative study on a good number of individuals with disabilities is planned to be conducted in near future.

**Comment #2:** *Additionally, the method presented eliminates the joystick as a means of control. However, the joystick also controls the speed of movement. There is a reference in the paper to further development of speed control. I believe this should be expanded upon, presenting at least a summary of the algorithm for gradual speed increase/decrease, as well as justification for choosing the optimal speed for long distances.*

**Response**: Thank you for your comment and recommendation. The major four wheelchair operations have been implemented in the paper. There is plan to develop more features under future works including the speed control, optimal speed selection. To implement these, we may have to use different new classes of hand gestures and establish new mathematical equations using positions and distances among the major landmarks and thresholds.

**Comment #3:** *The control part of the wheelchair motors is not described in terms of method and control parameters, especially in the implementation on a real wheelchair.*

**Response**: Thank you for your important remark and observation. The major four wheelchair control operations have been implemented and described in the paper. Upon satisfying the mathematical conditions, different gestures are recognized and different control operations are performed as per methodology where wheelchair motors are controlled with the support of Arduino-Uno and Raspberry pi. The description of the control circuit of the wheelchair motors has been included in revised manuscript. [Please see page 18 of the revised manuscript (under Section 4.1)].

The control circuit of the used smart wheelchair system consists of two motor-drivers combined with an Arduino Uno CPU and their connection to a DC power source. The system utilizes the MY1016Z2 motor as Fig. 7 illustrates. Its power rating is 250 Watts (0.33 Horsepower), and it runs at 24 V and 13.4 Amps. This motor can rotate in both clockwise and counterclockwise directions. Its rated load RPM is 400, and its no-load RPM is 3300. The motor driver model used here is the BTS7960. This driver's specifications are appropriate and meet the requirements. Compared to the conventional four relay control circuit, this motor driver offers a more convenient H-bridge control circuit. Each motor driver has 8 control pins including +5 V Vcc and GND; three pins of them are R_EN, RPWM, R_IS that are engaged in forwarding rotation, and the other three L_EN, LPWM, L_IS are engaged in the backward rotation. These 8 control pins are receiving signals from connected Arduino-Uno. Arduino is linked with Raspberry pi for receiving signals based on different hand gestures. Therefore, circumventing specific PWM values through Raspberry pi, the motors can be operated.

**Comment #4:** *Forward/backward/lateral movements by pointing with the index finger work for able-bodied individuals, but what is the error rate relative to the level of disability?*

**Response**: Thank you for your astute observation and comment. The Forward/backward/lateral movements by pointing with the index finger will work with same accuracy in similar way for the disabled persons who have at least the ability to move fingers of the hands to perform the gestures.

**Comment #5:** *It might have been beneficial to include a medical perspective, outlining the types and degrees of motor disabilities that can access this technique, as well as the method's advantages. Additionally, it could identify possible and potentially easier types of gestures.*

**Response**: Thank you for your insightful comment and observation. The disabled individuals who have the ability to move the fingers of the hand at least, can use the proposed technique. The technique has been tested in a real electric wheelchair operated by an actual disabled person also. A further detailed assessment and medical study with individuals with various degrees of motor disabilities couldn’t be performed due to permission issue.

**Comment #6:** *Although a known gesture recognition method is mentioned, it may be helpful to mention at least the main stages of the method.*

**Response**: Thank you for you observation and recommendation. The proposed gesture recognition technique has been clearly described in detailed steps in the paper. [Please see page 8 to page 16 of the revised manuscript (Section 3)]. The proposed technique utilizes a robust framework named MediaPipe Hands, for tracking the hands and fingers only which is a part of those steps. The short description about how MediaPipe Hands work, has been given in the revised manuscript also. [Please see page 9 of the revised manuscript (under Section 3.1)].
