# Supplementary material for: Developing a real-time hand-gesture recognition technique for wheelchair control
Source: PLoS One. 2025 Apr 24;20(4):e0319996. doi: 10.1371/journal.pone.0319996 (PMC12021275; doi:10.1371/journal.pone.0319996)
Supplement: Attachment — Submitted filename: Response_to_Reviewers_Comments_auresp_2.docx [file pone.0319996.s003.docx]

**RESPONSES TO REVIEWERS’ COMMENTS**

We would like to thank the reviewers for their careful read and constructive feedback on the previous draft entitled “**Developing a Real-Time Hand-Gesture Recognition Technique for Wheelchair Control**”. We have carefully considered their comments in preparing our revision, which has resulted in a manuscript that is clearer, more compelling and broader.

**Reviewers' Comments:**

....................................................................................................................................................................

**Reviewer #3:**

**Comment #1:** *Participants: The manuscript is missing Ethics Statement. As it involves a human participant.*

**Response:** Thank you for your valuable observation. Ethics Statement: Ethical approval for this experiment for testing the proposed technique by human participant, has been obtained from Institutional Review Board (IRB), BUET, Dhaka, Bangladesh (Ref/BUET/RISE/IRB/2025-01). [Please see Section 4.1 in page 19 of the revised manuscript]. The Ethical Approval letter also uploaded with manuscript.

**Ethics Statement:** Ethical approval for this experiment for testing the proposed technique by human participant, has been obtained from Institutional Review Board (IRB), BUET, Dhaka, Bangladesh (Ref/BUET/RISE/IRB/2025-01).

**Comment #2:** *The Table 1 have mistakes. Here are two numbers I found to be wrong. [22] and [23]' accuracies are wrong. Need revision.*

**Response:** Thank you for your precise observation. Sorry for the inconvenience The accuracies of mentioned research works have been revised in Table 1 accordingly. [Please see Table 1 under Related Work (Section 2) in page 7 of the revised manuscript].

**Comment #3:** *Table 1 has column for equipment and then list MATLAP, modified VGG-8 model and ROS as equipment. Need fix because they are not equipments.*

**Response:** Thank you for your incisive statement and observation. The ‘Major Equipment’ column of Table 1 has been revised for relevant research works, accordingly. Only actual equipment names were kept. [Please see Table 1 under Related Work (Section 2) in page 7 of the revised manuscript].

**Table 1. An Overview of Related Work.**

| **Existing Technique** | **Functionality** | **Limitations** | **Major Equipment** | **Accuracy** |
| --- | --- | --- | --- | --- |
| Gao et al. [19] | Hand gesture recognition | Requires hand raising, background complexity | Microsoft Kinect Camera, highly configured laptop | 10-100% (Depends on background complexity) |
| Oliver et al. [21] | Hand movement detection | Requires wearing hand band | Accelerometer, Joystick Manipulator | Actual accuracy is not measured |
| Sadi et al. [25] | Hand gesture recognition | Requires finger movement, doesn’t work well under daylight or if the background and skin tone match | RGB Camera, Raspberry Pi | 97.14% |
| Megalingam et al. [18] | Finger movement detection | The user must navigate the touchscreen with their hands or fingers and know how to operate the application. | Arduino Uno, HC05, Bluetooth, Android device | 98% |
| Desai et al. [20] | Eye movement detection | It’s a preliminary work to model the  control of a wheelchair, creates complication in real-time | HD Camera, Raspberry pi (planned) | Actual accuracy is not measured |
| Kutbi et al. [22] | Head movement Tracking | User’s head-motion is detected through a head-mounted egocentric camera, which is not user-friendly. | An egocentric camera, TI-TAN18CS, Arduino Mega | Accuracy not measured directly. Navigation time, number of collisions, survey comments were measured for a route in different modes. |
| Mahmud et al. [23] | Multi-modal wheelchair control mechanism | It’s integration of a joy stick with hand  gesture, eye gaze and head movement tracker. Not user-friendly, tracking sensors must be attached to the user's body | Raspberry pi, RGB camera and an accelerometer | Joystick: 94%  Hand gesture: 92%  Eye gaze: 86%  Head movement: 90%  Multiple: 94% |
| Tejonidhi et al. [24] | Eye pupil’s movement detection | Eye pupil is tracked by a camera and the system glows LEDs based on eye movements. The detection procedure is crucial for real-time applications | Web camera, Philips microcontroller | 70% to 90% |

**Comment #4:** *4. Figures are not places within the pdf but shown at the end. Need to be placed in the pdf.*

**Response:** Thank you for your important observation. As per the ‘Manuscript Organization’ section under ‘Submission Guidelines’ of PLOS One, only Figure captions were inserted immediately after the first paragraph in which the figure was cited. Figure files have been uploaded separately. The pdf was automatically built in system.

**Comment #5:** *5. Details about the 100 images collected with different size and background are missing. "The testing involved 100 samples for each hand gestures under different lightning conditions"*

**Response:** Thank you for your valuable comment and observation. Actually, for testing the proposed technique, for any specific gesture recognition, 100 samples were captured, which means, that gesture was performed 100 times, and for each time, hand landmarks’ co-ordinates are extracted, comparison done between distances and thresholds, and, if the mentioned logical conditions were met, then the relevant gesture was detected and a wheelchair operation was performed. The times these samples were taken and gestures were detected were counted. The detailed extracted data (co-ordinates of hand landmarks, distance matrix, detected gesture, thresholds) of 100 samples for each gesture have been included in ‘Data_Gesture_Recognition_extraction_outputs’ file accordingly.

**Comment #6:** *Big weakness this paper has is the small number of participants and the experiment is not clear. I understand it is difficult to hire people with difficulties. Thus, having one user from the target audience is a acceptable. However, authors can recruit able bodied people. Second point about the experiment. The author should design better experiments. Current experiment is testing the gesture while the wheelchair not moving and is designed only to test the ability of the system to read the gesture. But they did not test whether the user able to navigate within a specific route efficiently and safely. In conclusion, I suggest the authors hire more participants (not necessarily disabled) and design an experiment that test how long would it take the user to finish a navigation task and how comfortable and safe they are while navigating.*

**Response:** Thank you for your thoughtful observation. The proposed technique has been tested by an actual disabled person after taking proper consent. A qualitative study on a good number of individuals with disabilities and able-bodied people has been planned to be conducted some time soon, to validate the proposed technique more efficiently. Moreover, in order to test the actual usability of the proposed technique, an experimental evaluation in a specific test area with routes with blockages is planned to be performed in near future where different evaluation metrices such as: navigation time, number of collision, user survey will be evaluated for better assessment.
